# Catecholaminergic Modulation of Metacontrol Is Reflected by Changes in Aperiodic EEG Activity

**DOI:** 10.1093/ijnp/pyae033

**Published:** 2024-08-03

**Authors:** Yang Gao, Veit Roessner, Ann-Kathrin Stock, Moritz Mückschel, Lorenza Colzato, Bernhard Hommel, Christian Beste

**Affiliations:** School of Psychology, Shandong Normal University, Jinan, China; Cognitive Neurophysiology, Department of Child and Adolescent Psychiatry, Faculty of Medicine, TU Dresden, Dresden, Germany; Cognitive Neurophysiology, Department of Child and Adolescent Psychiatry, Faculty of Medicine, TU Dresden, Dresden, Germany; Cognitive Neurophysiology, Department of Child and Adolescent Psychiatry, Faculty of Medicine, TU Dresden, Dresden, Germany; School of Psychology, Shandong Normal University, Jinan, China; School of Psychology, Shandong Normal University, Jinan, China; Cognitive Neurophysiology, Department of Child and Adolescent Psychiatry, Faculty of Medicine, TU Dresden, Dresden, Germany

**Keywords:** Aperiodic EEG activity, catecholamines, methylphenidate, metacontrol

## Abstract

**Background:**

“Metacontrol” describes the ability to maintain an optimal balance between cognitive control styles that are either more persistent or more flexible. Recent studies have shown a link between metacontrol and aperiodic EEG patterns. The present study aimed to gain more insight into the neurobiological underpinnings of metacontrol by using methylphenidate (MPH), a compound known to increase postsynaptic catecholamine levels and modulate cortical noise.

**Methods:**

In a double-blind, randomized, placebo-controlled study design, we investigated the effect of MPH (0.5 mg/kg) on aperiodic EEG activity during a flanker task in a sample of n = 25 neurotypical adults. To quantify cortical noise, we employed the fitting oscillations and one over f algorithm.

**Results:**

Compared with placebo, MPH increased the aperiodic exponent, suggesting that it reduces cortical noise in 2 ways. First, it did so in a state-like fashion, as the main effect of the drug was visible and significant in both pre-trial and within-trial periods. Second, the electrode-specific analyses showed that the drug also affects specific processes by dampening the downregulation of noise in conditions requiring more control.

**Conclusions:**

Our findings suggest that the aperiodic exponent provides a neural marker of metacontrol states and changes therein. Further, we propose that the effectiveness of medications targeting catecholaminergic signaling can be evaluated by studying changes of cortical noise, fostering the idea of using the quantification of cortical noise as an indicator in pharmacological treatment.

**Significance Statement**

Methylphenidate (MPH) is a compound acting on the catecholaminergic system. In a neurotypical adult sample, we show that mechanisms of catecholaminergic modulation using MPH explain the causal link between metacontrol and aperiodic EEG patterns. The findings suggest that the effectiveness of medications targeting catecholaminergic signaling can be evaluated by studying changes of cortical noise, as reflected by aperiodic EEG activity. This suggests that quantifying cortical noise could serve as indicator in pharmacological treatment.

## INTRODUCTION

The planning, implementation, and execution of goal-directed human action is assumed to rely on cognitive control (Diamond, 2013). The concept of cognitive control is tailored according to the folk-psychological concept of willpower. Like willpower, the exertion of cognitive control is assumed to allow agents to stick to and follow their goals, ignore distracting information and temptations, and overcome obstacles. However, more recent considerations have acknowledged that this persistence function of cognitive control, as Hommel and Colzato (2017; Hommel, 2015) have called it, is only 1 side of the control coin. While persistence is essential for adaptive behavior under some circumstances, other circumstances call for the opposite: letting go, switching to other goals, making use of alternative opportunities, and being open to novel, unexpected information (Durstewitz et al., 2000; Goschke and Bolte, 2014; Cools, 2015). In line with others, Hommel and Colzato (2017) have called this the flexibility function of cognitive control. This raises the question of how people can find an appropriate situation-specific balance between persistence and flexibility, an ability that Hommel and Colzato refer to as metacontrol.

The present study was aiming to get more insight into the neurobiological underpinnings of metacontrol. Recent findings provided first evidence that engaging in persistence-heavy tasks or conditions is accompanied by a reduction of cortical variability. For instance, Zhang et al. (2023) found that a stimulus that signals a more persistent-heavy condition in a Go-Nogo task induces a significant increase of the aperiodic exponent in the EEG signal, which corresponds to a reduction of what one can consider cortical noise. This fits with findings of Zhang et al. (2022), who reported that greater variability of the resting-state BOLD signal is associated with worse performance in a persistence-heavy Stroop task but with better performance in a flexibility-heavy creativity task. This suggests that individual or condition-induced metacontrol biases toward persistence are associated with lesser cortical variability or cortical noise in the sense that experimental conditions that are calling for more persistence are associated with a lower degree of cortical noise.

Here we tested this hypothesis by using a drug to modulate the catecholaminergic system that is assumed to reduce cortical noise: methylphenidate (MPH), a compound that operates as a combined dopamine (DA) and norepinephrine (NE) transporter blocker, thereby increasing postsynaptic DA and NE levels (Volkow et al., 1999; Skirrow et al., 2015; Faraone, 2018). There is evidence that catecholaminergic signaling is a potentially suitable method for modulating metacontrol because of its effect on cortical noise. Catecholamines (DA and NE) modulate the signal-to-noise ratio of neuronal activity (Servan-Schreiber et al., 1990; Li et al., 2001; Cohen et al., 2002; Winterer and Weinberger, 2004; Aston-Jones and Cohen, 2005; Rolls et al., 2008; Kroener et al., 2009; Li and Rieckmann, 2014; Yousif et al., 2016; Ziegler et al., 2016; Vander Weele et al., 2018) by sharpening the distinction between irrelevant neural noise and relevant neural input, thereby increasing gain control processes, as indicated by a steeper slope of the sigmoid input-output function (Servan-Schreiber et al., 1990; Li et al., 2001; Warren et al., 2016). The slope indicates how quickly the output is changing with respect to the input. A steeper slope implies a more significant change in output for a small change in input, while a shallower slope indicates a more gradual change. That is, a steeper slope allows the system to amplify the signals from relevant inputs, making it easier to distinguish important information from neural noise. In contrast, a shallow slope may make the system more prone to being influenced by irrelevant neural noise. Accordingly, we expected that the intake of MPH leads to a reduction of cortical noise. Consistent with this idea, individuals with attention deficit hyperactivity disorder, who are suspected to suffer from a particularly strong flexibility bias (Colzato et al., 2022), are treated with MPH, which results in a steeper slope of the sigmoid input-output function but a less pronounced slope compared with neurotypical controls (when they are not taking medication; Pertermann et al., 2019). This implies that the effects of medication targeting catecholaminergic signaling can be assessed by observing changes in the slope, suggesting that the slope and the related quantification of cortical noise could serve as a valuable and innovative neurophysiological indicator in pharmacological treatment (Münchau et al., 2021).

In the present study, we used data from an existing EEG dataset (Bensmann et al., 2018) to test 2 hypotheses. First, we tested whether the administration of MPH reduces the cortical noise level of neurotypical participants. Our previous study (Zhang et al., 2023) successfully demonstrated changes in brain variability induced by particular stimuli. While this is encouraging, this success relies on active contributions from the participants: without appropriate instruction and task goals, the stimuli we presented would have been less likely to have the observed impact. If our neurobiological reasoning was correct, however, administering MPH should be equally potent in reducing cortical noise. To quantify cortical noise, we employed the fitting oscillations and one over f (FOOOF) algorithm (Donoghue et al. 2020), which allows the measurement of aperiodic activity in resting state and event-related EEG data, where a lower aperiodic exponent signifies a noisier and a higher exponent signifies a less noisy brain. The expected reduction of cortical noise might be general, as a kind of state effect, or specific, as a kind of task effect. Previous findings of Zhang et al. (2023) suggest that cortical noise reduction can be triggered by stimuli signaling a particular experimental condition, whereas drugs might have a more state-like impact. To disentangle these 2 possibilities, we used a task with conditions that differed with respect to their demands on metacontrol persistence (suggesting that they come with different degrees of cortical noise) and in which these demands differed unpredictably from one trial to another. We compared aperiodic exponents for the pre-stimulus interval (pre-trial period), during which participants could not yet know how persistence heavy the upcoming trial would be, with aperiodic exponents for the interval following the stimulus (within-trial period), during which participants did know the control demands of the condition. If the MPH manipulation mainly or exclusively affected the within-trial period, this would speak for a highly task-specific effect.

Second, we were interested to see whether the aperiodic exponent would follow the predicted pattern in a flanker task, which is commonly used in cognitive-control studies to assess inhibition and interference control, as well as selective or focused attention. In this task, a cognitive/response conflict was systematically induced in 2 ways. For one, the central target stimulus, an arrow pointing to the left or right to indicate the left or right key–pressing response, was accompanied by 2 irrelevant flankers above and below. When flankers and target point into the same direction (a condition we will refer to as flanker congruency), internal conflict should be less pronounced than when they point into different directions (flanker incongruency)—a condition that can be assumed to be more persistence heavy. If so, flanker incongruency should be accompanied by a stronger reduction of cortical noise, which in turn should result in a higher aperiodic exponent. For another, we presented a masked prime briefly before the target and the flankers. This prime consisted of an arrow pointing into the same or the opposite direction as the target, thus creating the 2 conditions prime congruency and prime incongruency, respectively. Again, the assumption was that prime incongruency would be the more persistence-heavy condition and thus induce a noise reduction resulting in a higher aperiodic exponent. As flanker effects are known to be much more robust than effects of masked primes (Boy et al., 2010; Stock et al., 2016, 2017), we were interested to see whether the aperiodic exponent would be sensitive to both manipulations of congruency and possibly the interactions between congruency and MPH.

## MATERIALS AND METHODS

### Participants

The study was based on an existing dataset (Bensmann et al., 2018, who were interested in and reported on the behavioral and the classical EEG findings of this study) collected from of N = 25 participants (mean age 23.92 years; SD 2.88; range 19–31 years; comprising 15 females). All participants were right-handed with normal or corrected-to-normal vision and without a history of neurological or psychiatric disorders. None of the participants reported regular drug and/or medication intake and did not consume caffeine on the day of investigation. Participants were recruited at TU Dresden. The present study was approved by the Psychology Research Ethics Committee of the TU Dresden. The original study was conducted in accordance with the Declaration of Helsinki and approved by the Ethics Commission of the Medical Faculty of the TU Dresden (EK420092015).

### MPH Administration

The study employed a within-subject design, with each participant undergoing 2 testing sessions (Bensmann et al., 2018). During 1 session, a single dose of MPH was administered, while a placebo was given in the other. The sequence of MPH and placebo administration was counter-balanced among participants and remained blind to the experimenter. The MPH dosage was individually calculated based on each participant’s body weight (0.50 mg/kg) at the onset of the first session. The investigation started approximately 2 hours after MPH administration because MPH plasma levels peak 1 to 3 hours after oral administration (Challman and Lipsky, 2000; Rösler et al., 2009). Participants were informed about the goals and procedure of this study and gave written consent (for details, see Bensmann et al., 2018).

### Task

**Figure 1.**
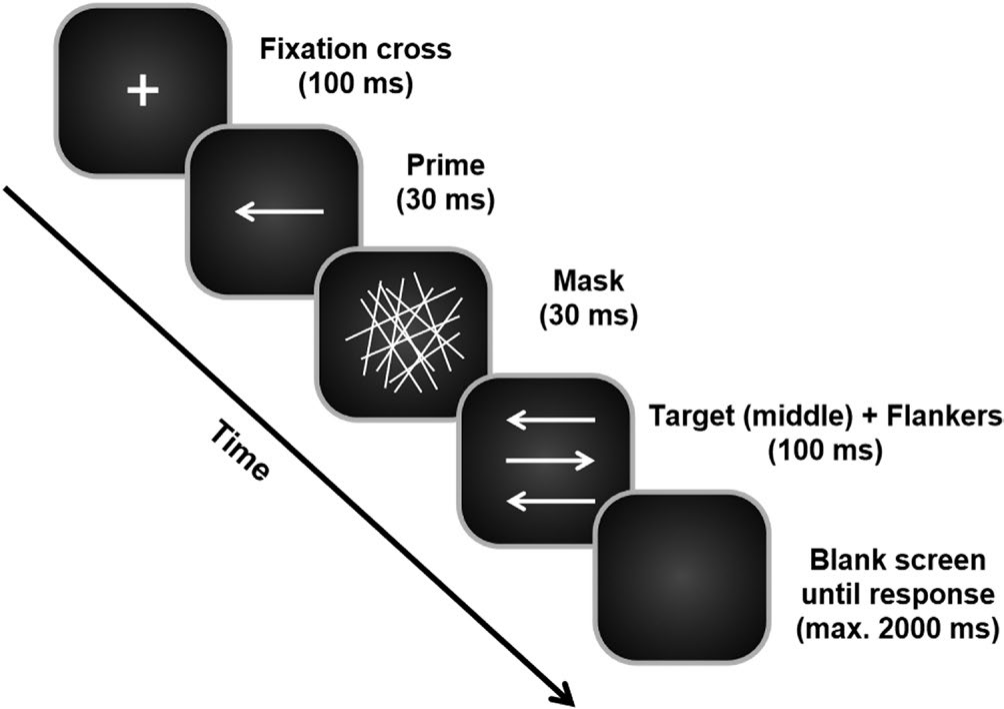
In the experimental paradigm utilized, each trial commenced with the display of a fixation cross lasting 100 milliseconds. Subsequently, a prime stimulus in the form of a central arrow was presented for a duration of 30 milliseconds, followed by the presentation of a mask array for an equal duration of 30 milliseconds. The target stimulus, consisting of a middle/central arrow along with flankers, was then presented for 100 milliseconds. Following the presentation of the target, the screen would turn black. To determine prime congruency, the direction of the primes in relation to the target was considered. Primes pointing in the same direction as the target were deemed prime congruent, while those pointing in the opposite direction were considered prime incongruent. Likewise, the congruency of the flankers was determined based on their direction in relation to the target: flankers pointing in the same direction as the target were classified as flanker congruent, whereas those pointing in the opposite direction were deemed flanker incongruent.

The task was based on a paradigm developed by Boy et al. (2010) and combines a target stimulus, a (presumably) subliminal prime, and consciously perceivable flankers. This allows the comparison of conflicts evoked by consciously and unconsciously processed sources (see Figure 1). The task was identical to the experimental setup of previous studies (Stock et al., 2016; Bensmann et al., 2020). Before the experiment, participants practiced the task. During the practice, feedback on response accuracy was provided, whereas no response feedback was included during the actual data collection phase of the experiment.

Each trial commenced with the presentation of a central white fixation cross on a black background for 100 ms (Figure 1), succeeded by the prime for 30 ms, a mask stimulus for another 30 ms, and then the target-flankers combination for 100 ms. The prime consisted of a single horizontal arrow pointing either right or left. The mask comprised an array of randomly arranged lines. The target, a single horizontal arrow pointing left or right, was located between 2 vertically aligned flanker stimuli. Participants were asked to identify the direction of the target arrow by pressing the right “Ctrl” key with the right index or middle finger for right-pointing target arrows, and the left “Ctrl” key with the left index or middle finger for left-pointing target arrows. Each trial ended with the participant’s response or when 2000 ms had elapsed after the onset of the target. The absence of a response categorized the trial as a “miss.” The response-stimulus interval between the participant’s response and the onset of the following trial was jittered between 1000 and 1200 milliseconds. Trials were deemed prime congruent (PC) when the prime and target arrows pointed in the same direction and prime incongruent (PI) otherwise. Similarly, trials were classified as flanker congruent (FC) or flanker incongruent (FI) based on the alignment of flankers and target arrows. This resulted in 4 distinct conditions: PC/FC, PC/FI, PI/FC, and PI/FI. In total, the task consisted of 384 trials equally divided into 4 blocks. All combinations of conditions were presented equally often and were randomized within each block. One session took approximately 15 minutes.

### EEG Recording and Processing

The data were recorded at the Cognitive Neurophysiology Lab at TU Dresden, Germany. During the task, the EEG activity was captured using QuickAmp amplifiers (Brain Products GmbH, Gilching, Germany) via 60 equidistant Ag/AgCl electrodes at a sampling rate of 500 Hz. All electrodes were referenced to Fpz. Electrode impedances were kept below 5 kΩ. The Brain Vision Analyzer 2.1 software package was employed for offline data preprocessing and the analysis of ERP data. Initially, raw EEG data were down sampled to 256 Hz, and a band-pass filter ranging from 0.5 to 20 Hz with a slope of 48 db/oct each was applied. Regular artifacts including muscle, eye, and cardiac interferences were then removed from all electrodes via independent component analysis (ICA). Irregular artifacts, such as technical noise, were manually inspected and removed from the raw data. Ultimately, previously discarded channels were interpolated using a spherical method. We used the following time windows: from −1200 to −200 milliseconds for pre-trial period and from 0–1000 ms for within-trial period. For more details about EEG recording and processing, please see the original published study (Bensmann et al., 2018).

### Parameterization of Spectral Data

EEG data were analyzed in 2 distinct time windows: from 0 milliseconds to 1000 milliseconds post-stimulus presentation (within-trial period) and from −1200 milliseconds to −200 milliseconds (pretrial period). Power spectral density (PSD) at each frequency was calculated using Welch method (0.25-second Hamming window, 50% overlap) (Welch, 1967). The calculation was implemented in Matlab using the “pwelch” function. The PSDs were estimated separately for each participant, electrode, condition, and both pre-trial and within-trial period. For estimating aperiodic activity, the power spectra were parameterized using the Python-based FOOOF toolbox (version 1.0.0; https://github.com/fooof-tools/fooof), which decomposes the signal into aperiodic and periodic components (for a detailed overview of this approach, see Donoghue et al., 2020), following the methodology of previous work (Adelhöfer et al., 2021). The FOOOF algorithm conceptualizes the power spectrum as a linear combination of aperiodic activity [*L*(*f*)] and periodic (oscillatory) activity [*G*n(*f*)]. Precisely, the model formula can be written as:


$$\begin{array}{l} PSD\left(\;f\right)=L\left(\;f\right)+\;\;\;\underset{n}{\sum }\;G_{n}\left(\;f\right) \end{array}$$

where *f* represents the frequency. The PSD is the linear combination of the aperiodic component, *L*(*f*), and n total Gaussians.

The aperiodic component is fit as a function across the entire fitted range of the spectrum. The function for the aperiodic component, *L*(*f*), is defined as:


$$\begin{array}{l} L\left(\;f\right)=b-log\left[f^{x}\right] \end{array}$$

where *b* represents the aperiodic offset reflecting the broadband power shift, and *x* denotes the aperiodic exponent, equivalent to the slope of the line fitted to the power spectrum in a log–log space.

The periodic (oscillatory) components are characterized as frequency regions of power over and above the aperiodic component. Each oscillatory component, also referred to as “peak,” is modeled with a Gaussian profile, defined by 3 distinct parameters. Each Gaussian fit can be modeled as:


$$\begin{array}{l} G_{n}\left(\;f\right)=a_{n}exp\left[-\;\;\;\frac{{\left(\;f-\;\;\;\mu_{n}\right)}^{2}}{2\sigma_{n}^{2}}\right] \end{array}$$

where *a*_*n*_ is the amplitude, *µ*_*n*_ is the center frequency, and *σ*_*n*_ is the bandwidth of each component.

To obtain a reliable estimation of the aperiodic component, the power spectra data were fitted over a broad frequency range of 3 to 35 Hz in accordance with recommendations in the FOOOF documentation. The FOOOF algorithm was configured with the following settings: {aperiodic mode = ‘fixed’, peak width limits = [2, 8], maximum number of peaks = 8, minimum peak height = 0.05, default settings otherwise}. The power spectra were fit for each electrode, each participant, each task condition, and each period. The average *R*^2^ of spectral fits for all participants (N = 25) was above 0.94.

### Aperiodic Exponent

The aperiodic parameters encompass both aperiodic exponent and aperiodic offset. The aperiodic exponent was found to be more theoretically transparent and particularly sensitive to index metacontrol states (Zhang et al., 2023), so our analysis focused on the exponent. Due to the absence of priori assumptions regarding the scalp distribution of the aperiodic neural activity, we derived the “global” aperiodic-only signal for each electrode and each participant (Hill et al., 2022). Initially, we averaged the exponent values across 60 electrodes for each participant (Hill et al., 2022) to discern the overall trend of variation. Subsequently, to investigate the distribution of the aperiodic components on the scalp, we conducted an extra cluster-based permutation test, resulting in statistically significant findings on a global scale. The nonparametric cluster-based permutation test is a method proposed to localize effects in space, frequency, and time while correcting for multiple comparisons in high-dimensional EEG/MEG data (Maris and Oostenveld, 2007). In this study, clusters were formed based on the adjacency of thresholded sample-level F-values (α = .005), with the sum of F-values within a cluster representing the cluster-level statistics. Significant clusters were determined based on 1000 Monte Carlo random sampling using a significance level of .05.

### Statistical Analysis

The behavioral and aperiodic exponent data were analyzed using SPSS software (IBM, version: 27.0). Only trials with correct responses were used for the further analysis of the aperiodic exponent. The *P* values were corrected using the Greenhouse-Geisser and Bonferroni methods whenever appropriate. For all descriptive statistics, the mean and the SEM are reported.

## Results

### Behavior

The behavioral data, as already reported in Bensmann et al. (2018), showed worse performance in reaction time and error rates with incongruent than with congruent flankers and with incongruent than with congruent primes. The error rates and the flanker-congruency effect were reduced under MPH, whereas the prime-congruency effect was not modified by MPH (see supplementary Material for details and statistics).

### Aperiodic Exponents (Brain-Wide)

**Figure 2.**
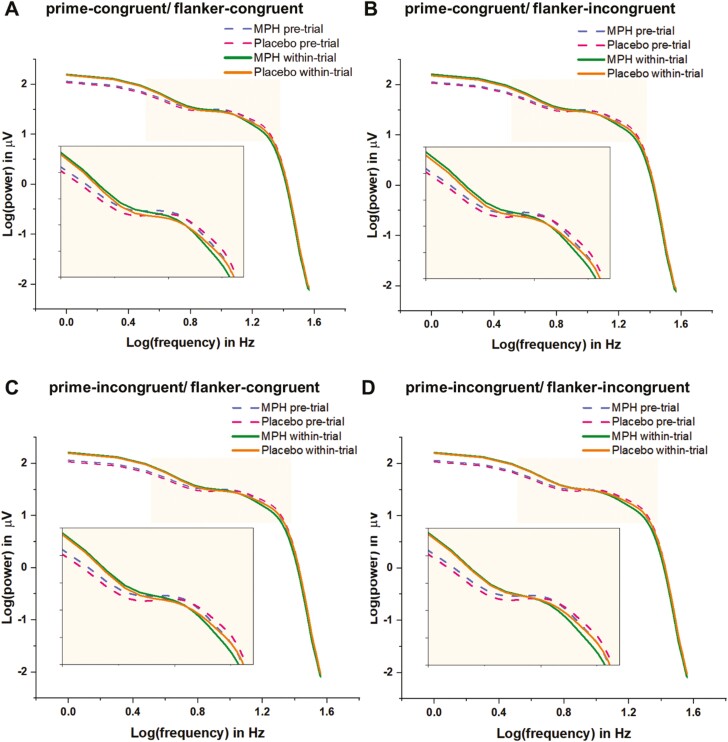
Log-log transformed power spectral density plot after averaging all electrodes and participants. (A) PSDs of MPH or placebo condition during pre-trial or within-trial period for the prime-congruent/flanker-congruent combination; (B) the prime-congruent/flanker-incongruent combination; (C) the prime-incongruent/flanker-congruent combination; and (D) the prime-incongruent/flanker-incongruent combination. The beige window in each panel was chosen to better visualize the differences between conditions.

PSD within a 3- to 35-Hz frequency range in log-log space is presented in Figure 2, illustrating the 4 different trial combinations (Figure 2A: prime congruent/flanker congruent, 2B: prime congruent/flanker incongruent, 2C: prime incongruent/flanker congruent, 2D: prime incongruent/flanker incongruent) in the MPH and placebo conditions during both pre-trial and within-trial periods. These PSDs are averaged across all electrodes and participants. The figure shows that the slope of the curve for the MPH group is steeper than the slope of the placebo group. Additionally, the curves are more pronounced during the within-trial phase compared with the pre-trial phase.

After calculating the average aperiodic exponent for all 60 electrodes for each participant, we ran a 2 (time: pre-trial, within-trial period) × 2 (drug: placebo, MPH) × 2 (prime: congruent, incongruent) × 2 (flanker: congruent, incongruent) ANOVA. The main effects of time (*F*_(1,24) _=_ _373.54, *P < *.001, *η*_*p*_^*2 *^=^* *^0.94, *BF*_*10 *_=_* *_1.58 × 10^14^), drug (*F*_(1,24) _=_ _17.88, *P < *.001, *η*_*p*_^*2 *^=^* *^0.427, *BF*_*10 *_=_* *_19.37), and prime-congruency (*F*_(1,24) _=_ _9.304, *P *=* *.006, *η*_*p*_^*2 *^=^* *^0.279, *BF*_*10 *_=_* *_8.55) were significant, indicating that the exponent was higher (i.e., noise was lower) in the within-trial period than in the pre-trial period (3.525 ± 0.044 vs 3.401 ± 0.044), under MPH administration than under placebo (3.528 ± 0.044 vs 3.399 ± 0.049), and with prime-incongruency than with prime-congruency (3.468 ± 0.044 vs 3.459 ± 0.044). Time interacted with flanker-congruency (*F*_(1,24) _=_ _7.45, *P *=* *.012, *η*_*p*_^*2 *^=^* *^0.237, *BF*_*10 *_=_* *_4.11) and with prime-congruency (*F*_(1,24) _=_ _14.77, *P < *.001, *η*_*p*_^*2 *^=^* *^0.381, *BF*_*10 *_=_* *_17.12).

**Figure 3.**
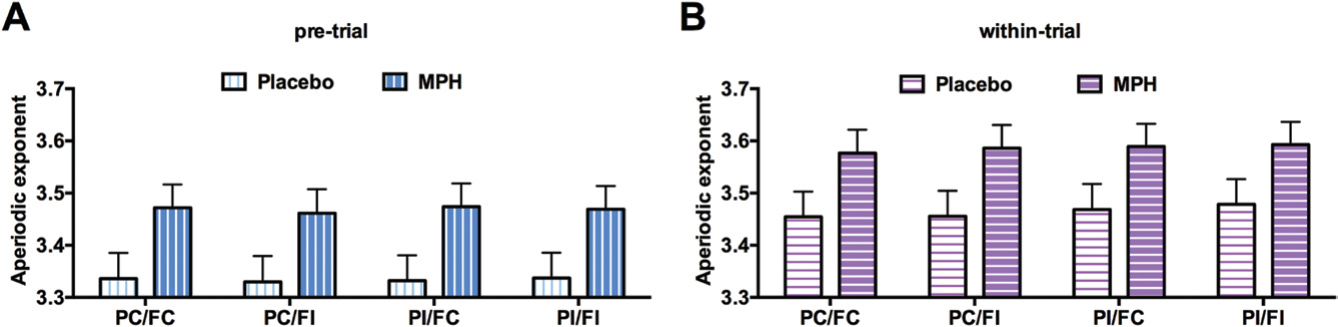
Sections A and B illustrate the aperiodic exponent under different task conditions, prime-congruent/flanker-congruent (PC/FC), prime-congruent/flanker-incongruent (PC/FI), prime-incongruent/flanker-congruent (PI/FC) and prime-incongruent/flanker-incongruent (PI/FI), with either placebo or MPH administration during pre-trial (A) or within-trial (B) periods. Error bars denote the SEM.

To disentangle this higher-order interaction involving time, we conducted separate ANOVAs for pre- and within-trial periods with the following factors: 2 (drug: placebo, MPH) × 2 (prime: congruent, incongruent) × 2 (flanker: congruent, incongruent). For the pre-trial data, this ANOVA yielded a significant MPH/placebo main effect (*F*_(1,24)_ = 20.178, *P < *.001, *η*_*p*_^*2*^ = 0.457, *BF*_*10*_ = 8.8), with a higher exponent under MPH administration (3.469 ± 0.045) than placebo administration (3.334 ± 0.049), thus indicating less aperiodic activity (less noise) in the MPH condition than in the placebo condition (Figure 3A). All other main effects and interactions were not significant (all F_(1,24)_ ≤ 2.231; *P* ≥ .148). For the within-trial data, the ANOVA uncovered significant main effects of MPH (3.586 ± 0.044)/placebo (3.464 ± 0.049) (*F*_(1,24)_ = 15.39, *P < *.001, *η*_*p*_^*2*^ = 0.391, *BF*_*10*_ = 12.85), of prime-congruency (*F*_(1,24)_ = 20.52, *P* < .001, *η*_*p*_^*2*^ = 0.461, *BF*_*10*_ = 207.48), and flanker-congruency (*F*_(1,24)_ = 6.082, *P *=* *.021, *η*_*p*_^*2*^ = 0.202, *BF*_*10*_ = 2.17) (Figure 3B). No interaction effect was found (all *F*_(1,24)_ ≤ 3.230; *P* ≥ .085). Figure 3A and B show that participants with MPH exhibited less aperiodic activity than placebo, both in the pre-trial and the within-trial period of the task. Please note that the exponent effects are typically very small numerically (Zhang et al., 2023; Jia et al., 2024; Pi et al., 2024), irrespective of significance.

### Aperiodic Exponents (Electrode-Specific)

**Figure 4.**
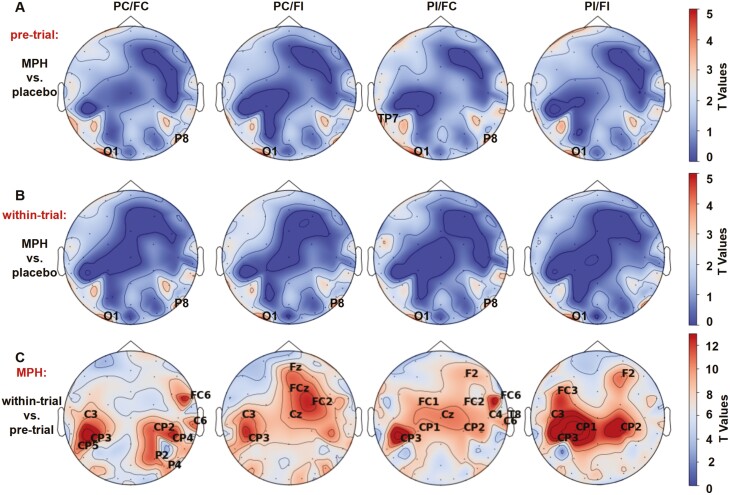
Scalp topographies (A) and (B) reveal electrode sites with a significant “MPH/placebo” main effect during pre-trial (MPH minus placebo) and within-trial (MPH minus placebo) periods across distinct task conditions. (C) Electrode sites with a significant main effect of time (within-trial minus pre-trial) in different task conditions. Labels indicate significant electrode clusters, with colors denoting the sum of cluster-level *t* values.

To identify electrodes that contributed to significant differences across our conditions, we assessed the scalp distribution of the exponent by means of a cluster-based permutation test (see Figure 4). Specifically, the cluster-based permutation 1-sample *t* test comparing MPH and placebo was separately performed for the 4 different combinations of prime congruency (PC, PI) and flanker congruency (FC, FI) for both the pre-trial period and the within-trial period.

**Figure 5.**
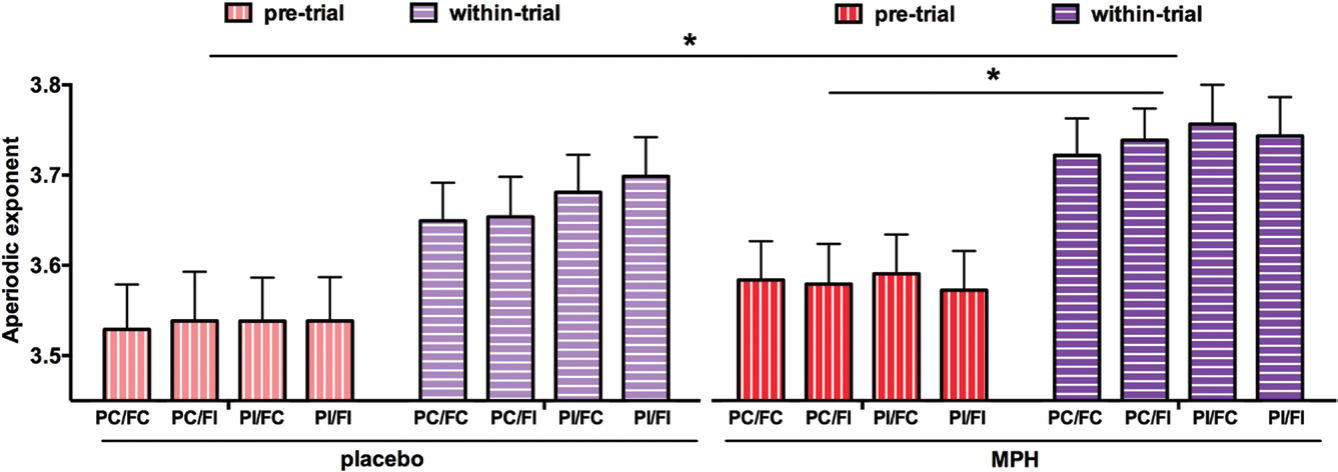
Behavioral results for the averaged aperiodic exponents of electrodes CP3 and C3 across distinct conditions. Bar plots represent the mean value of the corresponding condition and error bars represent SEM, *P < .05.

In the pre-trial period, the MPH/placebo effect was evident at O1 (*t*_(24)_ = 4.641; *P* = .01) and P8 (*t*_(24)_ = 3.522; *P* = .047) in the PC/FC condition; O1 (*t*_(24)_ = 4.379; *P* = .01) in the PC/FI condition; O1 (*t*_(24)_ = 4.922; *P* = .005), TP7 (*t*_(24)_ = 3.868; *P* = .031) and P8 (*t*_(24)_ = 3.761; *P* = .035) in the PI/FC condition; and O1 (*t*_(24)_ = 4.302; *P* = .011) in the PI/FI condition (Figure 5A). In the within-trial period, the MPH/placebo effect was evident at O1 (*t*_(24)_ = 4.107; *P* = .013) and P8 (*t*_(24)_ = 3.736; *P* = .037) in the PC/FC condition; O1 (*t*_(24)_ = 4.267; *P* = .012) and P8 (*t*_(24)_ = 4.011; *P* = .019) in the PC/FI condition; O1 (*t*_(24)_ = 3.980; *P* = .021) and P8 (*t*_(24)_ = 3.638; *P* = .038) in the PI/FC condition; and O1 (*t*_(24)_ = 4.354; *P* = .011) in the PI/FI condition (Figure 5B).

Electrodes exhibiting *t* > 10 were identified, signifying the most pronounced differences in these brain regions (Figure 4C).

Aperiodic exponents at CP3 and C3 exhibited the most significant changes before and during the trial. Therefore, focused statistical analysis was warranted for these 2 electrodes.

After averaging the aperiodic exponents of electrodes CP3 and C3 for each participant and each condition, we performed a 2 (time: pre/within-trial) × 2 (drug: placebo, MPH) × 2 (prime: congruent, incongruent) × 2 (flanker: congruent, incongruent) repeated-measures ANOVA. It identified main effects of time (*F*_(1,24)_ = 181.361, *P < *.001, *η*_*p*_^*2*^ = 0.987, *BF*_*10*_ = 1.87 × 10^11^), drug (*F*_(1,24)_ = 13.932, *P *=* *.001, *η*_*p*_^*2*^ = 0.367, *BF*_*10*_ = 29.21), and prime congruency (*F*_(1,24)_ = 27.132, *P < *.001, *η*_*p*_^*2*^ = 0.531, *BF*_*10*_ = 31.24) (Figure 5). Several interactions emerged: first, a time ×prime-congruency interaction (*F*_(1,24)_ = 16.082, *P < *.001, *η*_*p*_^*2*^ = 0.401, *BF*_*10*_ = 47.72) reflecting the fact that prime-congruency effects were restricted to the within-trial period (3.707 ± 0.043 vs 3.733 ± 0.046 for prime-congruent and prime-incongruent trials, respectively) but almost invisible even numerically in the pre-trial period (3.558 ± 0.048 vs 3.560 ± 0.046). Second, a prime-congruency × flanker-congruency interaction (*F*_(1,24)_ = 5.352, *P *=* *.030, *η*_*p*_^*2*^ = 0.182, *BF*_*10*_ = 1.07) was involved in a 3-way interaction with the factor drug (*F*_(1,24)_ = 4.374, *P *=* *.047, *η*_*p*_^*2*^ = 0.154, *BF*_*10*_ = 2.03). Separate ANOVAs for MPH and placebo including these factors 2 (time: pre/within-trial) × 2 (prime: congruent, incongruent) × 2 (flanker: congruent, incongruent) revealed that the prime-congruency × flanker-congruency interaction was significant only under MPH (*F*_(1,24)_ = 7.542, *P < *.05, *η*_*p*_^*2*^ = 0.239, *BF*_*10*_ = 2.57), but not under placebo administration (*F* < 1), indicating that the 2 congruency effects were additive under placebo but under additive under MPH (see Figure 5)—a pattern we will return to in the Discussion.

## Discussion

The aim of this study was to evaluate 2 hypotheses related to the neural underpinnings of metacontrol. Our first hypothesis was that MPH could be shown to reduce cortical noise under conditions with high demands on control. As explained in the introduction, there are reasons to assume that MPH increases metacontrol persistence, which in turn has been found to be associated with a reduction of cortical noise, as reflected in the aperiodic FOOOF exponent. Catecholamines are known to enhance the signal-to-noise ratio of neuronal activity (Servan-Schreiber et al., 1990; Li et al., 2001; Cohen et al., 2002; Winterer and Weinberger, 2004; Aston-Jones and Cohen, 2005; Rolls et al., 2008; Kroener et al., 2009; Li and Rieckmann, 2014; Yousif et al., 2016; Ziegler et al., 2016; Vander Weele et al., 2018) by improving the differentiation between irrelevant neural noise and relevant neural input (Servan-Schreiber et al., 1990; Li et al., 2001; Warren et al., 2016). We therefore predicted that the administration of MPH, a drug that acts as a dual DA/NE transporter blocker, results in a decrease of cortical noise. From a neural perspective, changes in aperiodic brain patterns are thought to indicate alterations in the balance between excitatory and inhibitory neural processes, referred to as the “E/I” ratio. A higher exponent value (indicating a steeper spectrum) usually signifies a dominance of inhibition over excitation, while a lower exponent suggests the reverse. Enhanced excitation levels have been linked to reduced synchronization between rhythmic brain oscillations and neuron firing, resulting in higher levels of neural noise that may disrupt neural communication (Voytek and Knight, 2015).

Indeed, as expected, MPH did increase the aperiodic exponent, suggesting that it was reducing cortical noise. On the one hand, it did so in a state-like fashion, as the main effect of the drug was visible and significant in both pre-trial and within-trial periods. On the other hand, however, the electrode-specific analyses showed that the drug also affects specific processes. In particular, it seems to dampen the downregulation of noise in more control-demanding conditions, which statistically led to an under-additive impact of the 2 types of incongruency under MPH. We can only speculate regarding the causes of this pattern but consider the existence of a biological limitation regarding the degree to which one can de-noise one’s brain a reasonable explanation. If so, we can tentatively conclude that each cue that is taken to indicate the challenge of cognitive control tends to induce the process of de-noising, which however hits a biological limitation at some point. With respect to attention deficit hyperactivity disorder, which is often treated by means of MPH, identifying limitations of that sort would be essential for predicting the possible impact of MPH-based interventions. In any case, as previously proposed by Münchau et al. (2021), the effectiveness of medications targeting catecholaminergic signaling can be evaluated by studying changes of cortical noise. Observing how cortical noise is altered in response to medication can potentially help to gain valuable insights not only into the impact of the medication on the underlying neurophysiological processes but also foster the idea of using the quantification of cortical noise as indicator in pharmacological treatment. Indeed, this might offer a unique way to assess and measure the effects of these medications on a neurological level, opening up new possibilities for understanding and optimizing treatment approaches with the end goal of developing a personalized approach in pharmacotherapy.

Our second hypothesis asked whether the previous observation of greater neural noise (or neural variability) reduction under more challenging control demands would generalize to a task in which 2 types of control demands were systematically varied: the congruency of irrelevant flankers with the target, and the congruency between the irrelevant prime and the target. While the flankers were clearly visible and easy to perceive, the prime was presented briefly and masked, suggesting that it was at least often invisible and unconsciously processed (Boy et al., 2010; Stock et al., 2016, 2017). Importantly, we obtained significant behavioral effects for both kinds of congruency, which serves as a manipulation check. Accordingly, we can be certain that both kinds of incongruency induced internal decision-making conflict, thus increasing the need for cognitive persistence (Boy et al., 2010; Stock et al., 2016, 2017). This was also reflected in the aperiodic exponents, which followed the pattern reported by Zhang et al. (2023). More specifically, incongruent conditions were accompanied by higher exponents than congruent conditions, and this effect was restricted to the within-trial period. This means that participants strategically reduced cortical noise upon the onset of stimuli that signal high demands on cognitive control in the upcoming trial. It is important to emphasize that participants reduced cortical noise from the pre-trial to the within-trial period in both congruent and incongruent conditions. This runs counter a characterization of our findings in terms of dual-route models or controlled vs automatic processing (e.g., Kornblum et al., 1990). Dual-route models would assume that controlled processing is required for incongruent, but not for congruent flankers, so that cognitive control would be restricted to processing incongruent flanker-target combinations. However, our findings suggest that control operations take place in both congruent and incongruent trials, as the increase of the exponent in both conditions demonstrates. However, the eventual metacontrol state that is implemented in these 2 conditions differs in terms of the degree of persistence, as the metacontrol model predicts.

Interestingly, the same outcome pattern was obtained for both flanker congruency and prime congruency, which implies that the difference between these conditions in terms of visibility, conscious awareness, and timing played no role with respect to noise reduction. In other words, every condition that is likely to challenge cognitive control seems to induce an adjustment of the cortical noise level, as far as assessed by our exponent, no matter how this challenge is created and whether the source of it can be consciously perceived. It is true that we did not explicitly check the degree to which participants were consciously aware of the existence and the nature of the primes they were presented with, so we have no direct evidence for the degree of awareness. It is also true that individuals differ with respect to the presentation parameters that are necessary to render particular stimuli subliminal (Boy et al., 2010; Stock et al., 2016, 2017). Nevertheless, given the parameters we used and the previous findings in studies using the same task (Boy et al., 2010; Stock et al., 2016, 2017), we think it is fair to say that on average, the primes were less likely to be consciously available than the flankers. Accordingly, finding comparable effects of prime and flanker congruency can be taken to suggest that conscious awareness of the source of conflict does not play a role for cortical noise adjustments, as reflected in the aperiodic exponent.

To conclude, the present study provides evidence that people respond to task- and stimulus-induced challenges of cognitive control by reducing their cortical noise down to a possible biological limit. MPH supports this de-noising process but also reduces the general noise level. From a metacontrol perspective, these findings provide converging evidence that the situational adjustment of metacontrol between the extreme poles of persistence and flexibility are associated with aperiodic activity, which decreases in the face of demands for more persistence. This fits with the idea that the aperiodic exponent provides a neural marker of metacontrol states and changes therein and with the possibility that metacontrol changes are neurally achieved by moderating the brain’s noise level.

## Supplementary Material

pyae033_suppl_Supplementary_Materials

## Data Availability

All data can be obtained from the corresponding author upon reasonable request. For further questions, please contact Yang Gao (gaoyang.2017@qq.com).
