# Supplementary material for: Catecholaminergic Modulation of Metacontrol Is Reflected by Changes in Aperiodic EEG Activity
Source: Int J Neuropsychopharmacol. 2024 Aug 3;27(8):pyae033. doi: 10.1093/ijnp/pyae033 (PMC11348007; doi:10.1093/ijnp/pyae033)
Supplement: pyae033_suppl_Supplementary_Materials [file pyae033_suppl_supplementary_materials.docx]

**Behavioral Results**

**Error Rates**

The mean percentage of incorrect responses was analyzed by performing a repeated measures ANOVA with three within-subject factors of drug (MPH, placebo), prime (congruent, incongruent), and flanker (congruent, incongruent). Results showed significant main effects of all three factors: MPH/placebo (*F*_(1,24)_=9.29, *p*=0.006, *η_p_^2^*=0.279, *BF_10_*=8.79), prime-congruency (*F*_(1,24)_=47.27, *p*<0.001, *η_p_^2^*=0.663, *BF_10_*=35850), and flanker-congruency (*F*_(1,24)_=32.96, *p*<0.001, *η_p_^2^*=0.579, *BF_10_*=1687) (Footnote 1), as also reported by Bensmann et al. (2018). Participants made more errors under placebo than MPH administration, in prime-incongruent than prime-congruent conditions, and in flanker-incongruent than flanker-congruent conditions. More interestingly, MPH/placebo and flanker-congruency showed a significant interaction (*F*_(1,24)_=5.36, *p*=0.029, *η_p_^2^* =0.183, *BF_10_*=1.1) (Footnote 1). Further analyses revealed that the effect of flanker-congruency was significant in both the placebo (*t*_(24)_=5.316, *p*<0.001, *d*=1.063, *BF_10_*=2443.249) and the MPH condition (*t*_(24)_=4.029, *p*<0.001, *d*=0.806, *BF_10_*=130.290), indicating worse performance in the incongruent condition (5.98%±0.875) than in the congruent condition (3.316%±0.551) under placebo administration and in under MPH administration (3.747%±0.523 vs. 2.249%±0.362, respectively; see Figure 1SA). Yet, the flanker-congruency effect (i.e., flanker-incongruent minus flanker-congruent) was larger under placebo administration (2.66%±2.50) than MPH administration (1.49%±1.86)(*t*_(24)_=2.31, *p*=0.030, *d*=0.46, *BF_10_*=3.85) (Figure 1SB). No significant interaction was obtained between MPH/placebo and prime-congruency (*F*_(1,24)_=1.98, *p*=0.172, *η_p_^2^*=0.076, *BF_10_*=0.704).

**Response Times (RTs)**

The analysis of RTs in correct trials revealed main effects of prime-congruency (*F*_(1,24)_= 287.34, *p*<0.001, *η_p_^2^*=0.923, *BF_10_*=1.29×10^12^) and flanker-congruency (*F*_(1,24)_=86.45, *p*<0.001, *η_p_^2^*=0.783, *BF_10_*=5.02×10^6^), indicating worse performance in incongruent than in congruent conditions, respectively. The MPH/placebo factor did not produce a main effect (*F*_(1,24)_=0.806, *p=*0.378, *η_p_^2^*=0.032, *BF_10_*=1.87×10^11^) but was involved in an interaction with flanker-congruency (*F*_(1,24)_=6.90, *p*=0.015, *η_p_^2^*=0.223, *BF_10_*=0.77)(Figure 1SC). Simple effect analyses indicated that flanker-congruency was significant under both MPH (*t*_(24)_=5.71, *p<*0.001, *d*=1.142, *BF_10_*=6027; 436.81 ±11 ms vs. 451.83 ±10.9 ms for congruent and incongruent trials, respectively) and placebo conditions (*t*_(24)_=9.08, *p<*0.001, *d*=1.82, *BF_10_*=7.58×10^6;^ 440.50±10.38 ms vs. 463.67±10.38 ms). Posthoc tests indicated that the flanker-congruency effect was larger under placebo (23.18 ms±12.81) than MPH administration (15.03 ms±12.93) (*t*_(24)_=2.63, *p*=0.015, *d*=0.53, *BF_10_*=6.84)(Figure 1SD). No significant interaction was obtained between MPH/placebo and prime-congruency (*F*_(1,24)_=1.16, *p*=0.293, *η_p_^2^*=0.046, *BF_10_*=0.658).


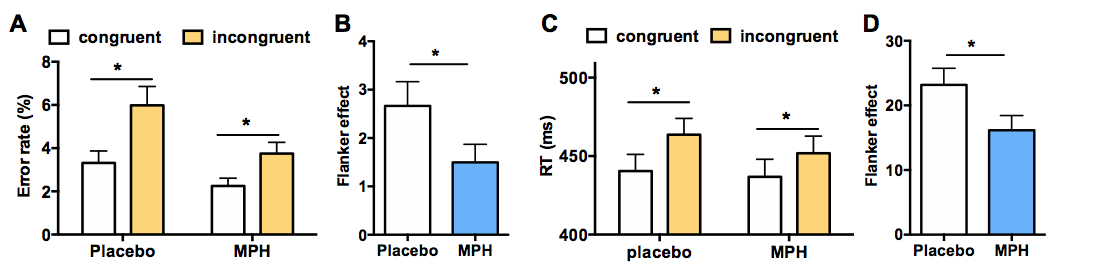


**Figure 1S.** (A) Error rates data are displayed for the various conditions. (B) The flanker-congruency effect (i.e., incongruent minus congruent) is shown for the placebo and the methylphenidate (MPH) conditions. (C) Reaction time (RT) data are shown for the different conditions, and (D) the flanker-congruency effect (i.e., incongruent minus congruent) is shown for placebo and MPH conditions. Significant results (*p* <0.05) are denoted with an asterisk. Error bars represent the SEM to indicate variability.

FOOTNOTE 1

Please note that, in contrast to Bensmann et al. (2018), we report Error Rates instead of Accuracy. While this might have led to slight differences in the rounding of the third number of the F values (as compared to Bensmann et al., 2018), it facilitates comparisons between error and reaction time data.
